# Traditionally Used *Sideritis cypria* Post.: Phytochemistry, Nutritional Content, Bioactive Compounds of Cultivated Populations

**DOI:** 10.3389/fphar.2020.00650

**Published:** 2020-05-12

**Authors:** Krystalia Lytra, Ekaterina-Michaela Tomou, Antonios Chrysargyris, Chryssoula Drouza, Helen Skaltsa, Nikolaos Tzortzakis

**Affiliations:** ^1^Department of Pharmacognosy & Chemistry of Natural Products, School of Pharmacy, National & Kapodistrian University of Athens, Athens, Greece; ^2^Department of Agricultural Sciences, Biotechnology and Food Science, Cyprus University of Technology, Lemesos, Cyprus

**Keywords:** *Sideritis cypria*, cultivation, infusions, flowers, leaves, melittoside, leonoside A, lamalboside

## Abstract

*Sideritis* species are recognized as important medicinal plants and their commercial demand is continuously on the rise both in the European and in the global market. Consequently, the cultivation of *Sideritis* species has been occurred to successfully meet the need for mass production of high-quality plant material. The present study was undertaken in order to investigate the chemical composition of cultivated *S. cypria*. Infusions of flowers and leaves were prepared separately, according to the European Medicine Agency (EMA) monograph. The infusion of the flowers revealed the presence of four flavones, isoscutellarein-7-O-[6′″-O-acetyl-β-D-allopyranosyl-(1→2)-β-D-glucopyranoside, its 4′-O-methyl-derivative, 4′-O-methyl-hypolaetin-7-O-[6′″-O-acetyl-β-D-allopyranosyl-(1→2)-β-D-glucopyranoside, and isoscutellarein-7-O-[6′″-O-acetyl-β-D-allopyranosyl-(1→2)]-6″-O-acetyl-β-D-glucopyranoside; four phenylethanoid glucosides, acteoside, leucosceptoside A, lamalboside, and leonoside A; one iridoid, melittoside, and one phenolic acid, chlorogenic acid, while the infusion of the leaves of the same population afforded the same first two flavones; five phenylethanoid glucosides, acteoside, leucosceptoside A, lavandulifolioside, leonoside A, and lamalboside; melittoside and chlorogenic acid. The structural elucidation of the isolated compounds was undertaken by high-field NMR spectroscopy. Moreover, the essential oils of the flowers and leaves were studied by GC-MS, separately. In addition, the mineral, bioactive compounds, protein and carbohydrate contents were evaluated for both plant materials.

## Introduction

*Sideritis* species (Lamiaceae) have been used as traditional medicine herbs for thousands of years (González-Burgos et al., 2011) and the last 30 years safe use (including 15 years in the EU) with well-defined posologies and mode of preparation (EMA, 2015). Nowadays, the infusion of *Sideritis scardica* Griseb.; *Sideritis clandestina* (Bory & Chaub.) Hayek; *Sideritis raeseri* Boiss. & Heldr.; and *Sideritis syriaca* L., has been listed by European Medicine Agency (EMA) as a traditional medicine for the relief of mild gastrointestinal discomfort and against the common cold (EMA, 2015). Previous detailed studies underlie the important pharmacological activities of the genus such as the antioxidant, anti‐inflammatory, antivirus, anticancer, hepatoprotective, antispasmodic, analgesic, neuroprotective activity, as well as its great effectiveness against diseases related to the central nervous and to the urinary system (Kirimer et al., 2004; González-Burgos et al., 2011; EMA, 2015; Hofrichter et al., 2016; Deveci et al., 2017; Aneva et al., 2019). Indeed, a clinical study that carried out by Wightman et al. (2018), showed that *S. scardica* (Greek mountain tea) improved the aspects of cognitive function and mood in a group of healthy, older adults. Therefore, these plants have been a subject of intensive phytochemical research and are characterized mainly by the presence of terpenes, flavonoids, phenylethanoid glucosides, phenolic acids, and essential oil (González-Burgos et al., 2011; EMA, 2015).

The genus *Sideritis* L. comprises around 150 species (Aneva et al., 2019); among them, the endemic species *S. cypria* Post. is a perennial herb belonging to the section *Empedoclia* Rafin., 60 cm high with bright yellow flowers, growing at altitude 300–925 m in Pentadactylos Mountains in Northern Cyprus (Meikle, 1985; Yildiz and Gücel, 2006; Tsintides et al., 2007). In particular, three *Sideritis* species have been found in Cyprus; *S. curvidens* Stapf, *S. perfoliata* L., and the endemic *S. cypria* Post (Hanoğlu et al., 2020). Traditionally, the infusion of *S. cypria* is locally used as diaphoretic, tonic, as well as against stomach disorders, headache and common cold (Yöney et al., 2010; Karousou and Deirmentzoglou, 2011; Hanoğlu et al., 2020). Hanoğlu et al. (2016) reported the antimicrobial efficacy of essential oils derived by *S. cypria*, and suggested to be used as a new medicinal resource particularly against *C. albicans* and Gram-positive bacteria.

However, the secondary metabolites of the traditional infusion of this plant are still undershadowed. The literature survey revealed only two publications concerning the chemical characterization of wild harvested *S. cypria* of its essential oil (Hanoğlu et al., 2016) and extracts (Hanoğlu et al., 2020). Therefore, for the purpose of this study, we investigated and compared for the first time the chemical composition of the traditional infusions of different plant parts, leaves and flowers, of cultivated *S. cypria*. In parallel, the essential oils and the mineral, bioactive compounds, protein, and carbohydrate contents of the flowers and the leaves of cultivated populations have been studied.

## Materials and Methods

### Plant Material

**Figure 1:**
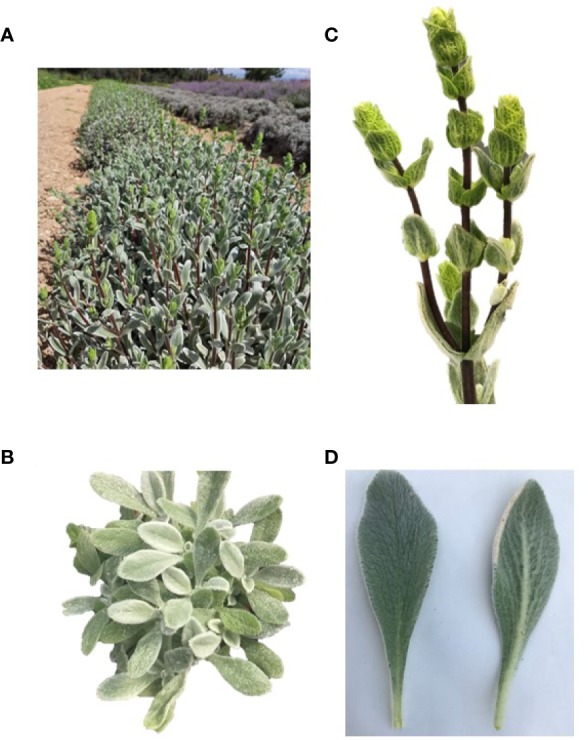
*Sideritis cypria*, **(A)** cultivated crop, **(B)** plant, **(C)** flower, and **(D)** upper and bottom leaf area.

*Sideritis cypria* was collected from the Cypriot National Agricultural Department, on June 2019. Species seeds are kept at the Agricultural Research Institute, national gene bank (Accession number: ARI02415), collected in 2018 (Universal Transverse Mercator-UTM with latitude 546102; longitude 3904418, altitude 640). This is the first registered time that the plant has been cultivated, and plants from the first (mother) plantation were collected at their flower stage, for this analysis. Plantation was established at 2018, frequently irrigated (~ weekly/biweekly during irrigation period) and common fertilizers applied, with 20-10-10 once a year as basic fertilizer and 19-19-19 every second month as fertigation. Representative images of the different plant parts are presented in **Figure 1**.

### Mineral Content and Nutritional Value

In the present study we use the word “flowers” in a wide sense which is usual in pharmacy. In this case we refer as "flowers" the whole inflorescence, including corollas, calyces, bracts and axis. Thus, we do not use “flowers” in its strict botanical sense. Aerial parts of the plants were separated into leaves and flowers (including corollas, calyces and bracts) and dried until constant weight at 65°C, using an air-drying oven. Dried tissue was then milled to pass a 0.42 sieve and ashed in a furnace (Carbolite, AAF 1100, GERO, Germany) at 450°C for 6 h. Nitrogen was determined using the Kjeldahl method (BUCHI, Digest automat K-439 and Distillation Kjelflex K-360, Switzerland). For the determination of minerals, tissue was acidified with 2 N hydrochloric acid (HCl). Phosphorus (P) was assayed with the molybdate/vanadate method while potassium (K), calcium (Ca), sodium (Na), magnesium (Mg), copper (Cu), zinc (Zn), and iron (Fe) were determined using an atomic absorption spectrophotometer (PG Instruments AA500FG, Leicestershire, UK) as described previously (Chrysargyris et al., 2019a). The nutritional value of leaves and flowers was determined according to the methods described by AOAC (AOAC, 2016). The protein content (in dry weight; d.w.) was determined by using the Kjeldahl method (N × 6.25), petroleum ether Soxhlet extraction was used for total fat determination, and tissue incineration (600°C) for moisture. The total content of carbohydrates was determined by difference, and the energetic value was calculated using the following formula: Energy (kcal/100 g d.w.) = 4 × (g protein/100 g d.w. + g carbohydrate/100 g d.w.) + 9 × (g fat/100 g d.w.). Results were expressed in g/100 g d.w. Four replicates were analyzed for each plant part, while each replicate was a pool of three different plants (**Table 1**).

**Table 1:** Mineral analysis and nutritional value of aerial plant tissue, leaves, and flowers.

	Minerals			Nutritional Value	
	Flowers	Leaves		Flowers	Leaves
N (g/kg Dw)	14.43 ± 0.15a	12.91 ± 0.20b	Dry Matter (%)	28.75 ± 039a	29.19 ± 0.14a
K (g/kg Dw)	15.73 ± 0.40b	28.40 ± 0.14a	Moisture (%)	71.24 ± 0.39a	70.80 ± 0.14a
P (g/kg Dw)	4.00 ± 0.08a	3.49 ± 0.04b	Ash (%)	6.47 ± 0.06b	17.21 ± 0.20a
Mg (g/kg Dw)	2.54 ± 0.05a	2.04 ± 0.13b	Protein (%)	9.02 ± 0.09a	8.06 ± 0.12b
Ca (g/kg Dw)	14.65 ± 0.47b	20.13 ± 0.49a	Total Fats (%)	3.85 ± 0.06a	1.78 ± 0.87b
Na (g/kg Dw)	0.55 ± 0.01b	2.01 ± 0.14a	Carbohydrates (%)	80.65 ± 0.18a	72.90 ± 1.32b
Cu (mg/kg Dw)	64.95 ± 8.63b	113.09 ± 7.60a	Energy (kcal/100g Dw)	393.35 ± 0.29a	335.88 ± 18.11b
Zn (mg/kg Dw)	62.12 ± 3.77a	59.79 ± 1.67a			
Fe (mg/kg Dw)	153.77 ± 14.96b	398.87 ± 20.23a			

Data are presented as the mean of four replicates ± SE. Values in column for each extraction method followed by the same letter are not significantly different, P ≤ 0.05.

### Bioactive Compounds of Plant Extracts

Both the infusion and the decoction of leaves and flowers were used for the estimation of the total phenolic and flavonoid content, as well as the evaluation of bioactive compounds of the plant (**Table 2**). The extracts were diluted with 50% methanol to reach a concentration of 2 mg/ml. The phenolic content was determined according to the Folin-Ciocalteu method. The reaction mixture consisted of 0.5 ml of each extract (2 mg/ml), 0.5 ml of Folin Ciocalteu reagent, and 5 ml of water. The reaction was incubated for 1 h before absorbance was measured at 765 nm (Goulas et al., 2014). Results were expressed as mg of gallic acids equivalents per g of dry material. Total flavonoids content was estimated as described by Chrysargyris et al. (2016), using the aluminum chloride colorimetric method, and results were expressed as mg of rutin equivalents per g of dried tissue.

**Table 2:** Total phenolics and flavonoids content and bioactive compounds of infusion and decoction of *S. cypria*.

		Total phenolics (mg GAE/g Dw)	DPPH (IC50 mg/mL)	FRAP (mg TROLOX/g Dw)	Flavonoids (mg RUTIN/g Dw)
Infusion	Leaves	3.022 ± 0.039b	792.992 ± 2.096a	75.926 ± 1.494b	7.721 ± 0.164b
	Flowers	8.019 ± 0.173a	267.891 ± 1.630b	199.374 ± 1.364a	32.004 ± 0.169a
Decoction	Leaves	1.396 ± 0.018b	605.536 ± 9.145a	98.418 ± 3.775b	3.604 ± 0.034b
	Flowers	3.972 ± 0.031a	404.995 ± 6.884b	159.729 ± 3.073a	9.961 ± 0.188a

Data are presented as the mean of four replicates ± SE. Values in column for each extraction method followed by the same letter are not significantly different, P ≤ 0.05.

Extracts bioactivity was evaluated by the DPPH (2,2-diphenyl-1-picrylhydrazyl) and the ferric reducing antioxidant power (FRAP) assays. Serial dilutions of the extracts (0–1,000 μg/ml) were incubated with 0.3 mmol/L of DPPH for 30 min, and absorbance was measured at 517, as described by Goulas et al. (2014). The % of scavenging activity was determined using the formula: scavenging activity %=100-(Absorbance _samples_-Absorbance _blank_)100/Absorbance _control_. IC50 values are the concentration of each extract that has the 50% of the antioxidant activity. The FRAP assay was also tested, as described by Chrysargyris et al. (2016), using serial dilutions of Trolox [(±)-6-hydroxy-2,5,7,8-tetramethylchromane-2-carboxylic acid], as the reference standard. Results were expressed as mg Trolox per g of dried weight. All samples were tested in four replicates.

### Essential Oils Extraction and Analysis

Aerial plant parts (leaves and flowers, separately) were placed in an air-drying oven at 42°C, for 48 h, until constant weight. Hydrodistillation was adopted for the extraction of the essential oils, using Clevenger apparatus. The duration of the extraction was 3 h for each sample, while four samples (pool of three plants per sample) were subjected for extraction and then for oil analysis. The essential oil yield was measured as μl of oil per 100 g of dried tissue.

The analysis of the essential oil components was carried out using a Shimadzu GC2010 gas chromatograph interfaced with a Shimadzu GC/MS QP2010plus mass spectrometer. A sample of each essential oil (1 μl) diluted in ethyl acetate (1:1,000) was injected in the autosampler, in split mode (1:20). The injector temperature was set at 230°C and the column temperature was programmed to rise from 60°C to 240°C at a rate of 5°C/min, with a 5 min hold at 240°C. Compound separation was performed on a Zebron ZB-5 (Phenomenex, USA) capillary column (0.25 μm x 30.0 m x 0.25 mm). Helium was used as the carrier gas with a flow set at 1.03 ml/min. Electron mass spectra with ionization energy of 70 eV was recorded at 35–400 m/z. The identification of oil components (**Table 2**) was assigned by comparison of their retention indices relative to (C_8_–C_20_) n-alkanes with those of literature or with those of authentic compounds available in our laboratory, by matching their recorded mass spectra with those stored in the NIST08 mass spectral library of the GC–MS data system and other published mass spectra (Adams, 2012).

### Isolation and Identification of Secondary Metabolites of Flowers and Leaves Infusions

#### General Experimental Procedures

1D- and 2D-NMR spectra were recorded in CD_3_OD on Bruker DRX 400 instrument at 295 K. Chemical shifts are given in ppm (δ) and were referenced to the solvent signals at 3.31 and 49.0 ppm. COSY (COrrelation SpectroscopΥ), HSQC (Heteronuclear Single Quantum Correlation), HMBC (Heteronuclear Multiple Bond Correlation), and NOESY (Nuclear Overhauser Effect SpectroscopY) (mixing time 950 ms) experiments were performed using standard Bruker microprograms. The [α]_D_ values were obtained in CH_3_OH on Perkin Elmer 341 Polarimeter. UV-Vis spectra were recorded on a Shimadzu UV-160A spectrophotometer, according to Mabry et al. (1970). Column chromatography (CC): silica gel (Merck, Art. 9385, Darmstadt, Germany), gradient elution with the solvent mixtures indicated in each case. Preparative‐thin‐layer chromatography (TLC) plates pre‐coated with silica gel (Merck, Art. 5721). Fractionation was always monitored by TLC silica gel 60 F‐ 254, (Merck, Art. 5554) and cellulose (Merck, Art. 5552) with visualization under UV (254 and 365 nm) and spraying with vanillin‐sulphuric acid reagent and with Neu’s reagent (Neu, 1957) for phenolic compounds. In the whole process, all the obtained infusions, fractions, and isolated compounds were evaporated to dryness in vacuum under low temperature and then were put in activated desiccators with P_2_O_5_ until their weights had stabilized, in order to eliminate the moisture from the samples that might influence pre‐saturation performance and then lead to an intense water signal in the ^1^H‐NMR spectra, making it difficult to observe near signals.

#### Fractionation and Isolation Procedure

Infusions of cultivated *S. cypria* Post were prepared based on the monograph of EMA, namely, 4.0 g of air-dried flowers and 4.0 g of air-dried leaves were separately dropped into 200 ml boiled distilled water each for 5 min, then were filtrated and concentrated to dryness. The infusion of flowers (1.1 g) was fractionated by CC over silica gel using as eluent mixtures of CH_2_Cl_2_:MeOH:H_2_O 9.9:0.1:0.01-0:1:0 to yield finally 27 fractions (A-Z_1_). Fractions J and K were combined (6.0 mg; eluted with CH_2_Cl_2_:MeOH:H_2_O 8:2:0.2-7.8:2.2:0.2) and afforded compound **4** (4.1 mg). Fraction N (eluted with CH_2_Cl_2_:MeOH:H_2_O 7.8:2.2:0.2) yielded compound **1** (3.3 mg). Fraction Q (10.2 mg; eluted with CH_2_Cl_2_:MeOH:H_2_O 7.5:2.5:0.2) was further purified by preparative TLC plates pre‐coated with silica gel and gave compounds **3** (1.7 mg) and **6** (3 mg). Combined fractions R, S, T, and U (108.0 mg; eluted with CH_2_Cl_2_:MeOH:H_2_O 7.5:2.5:0.25-6.8:3.2:0.32) were further fractionated by CC over silica gel using as eluent mixtures of CH_2_Cl_2_:MeOH:H_2_O 9.7:0.3:0.03-0:1:0 and afforded compound **1** (0.7 mg), a mixture of compounds **2** and **6** (0.7 mg), as well as compounds **5** (3.4 mg) and **8** in mixture with free sugars (5.2 mg). Compound **8** was further purified by preparative TLC plates pre-coated with silica gel and gave pure compound **8** (2.4 mg). Fraction Y (58.8 mg; eluted with CH_2_Cl_2_:MeOH:H_2_O 6:4:0.4) was subjected to CC over Sephadex (MeOH:H_2_O 100:0-90:10) and yielded compounds **9** (5.1 mg), **10** (15.3 mg) and **11** (1.6 mg).

The infusion of leaves (0.7 g) was fractionated by CC over silica gel using as eluent mixtures of CH_2_Cl_2_:MeOH:H_2_O 9.9:0.1:0.01-0:1:0 to afford finally 23 fractions (A–W). Fraction I (0.9 mg; eluted with CH_2_Cl_2_:MeOH:H_2_O 7.8:2.2:0.2) was identified as compound **1** (0.9 mg). Fractions K and M were eluted with CH_2_Cl_2_:MeOH:H_2_O 7.5:2.5:0.2 and gave compounds **2** (0.7 mg) and **6** (1.2 mg), respectively. Fraction Q (56.3 mg; eluted with CH_2_Cl_2_:MeOH:H_2_O 7:3:0.3-6.8:3.2:0.32) was applied to CC over silica gel using as eluent mixtures of CH_2_Cl_2_:MeOH:H_2_O 9.9:0.1:0.01-0:1:0 and afforded compounds **5** (2.5 mg) and **8** (0.7 mg). Fraction S was eluted with CH_2_Cl_2_:MeOH:H_2_O 6.5:3.5:0.35 and yielded compound **7** (9.0 mg). Fraction U (61.0 mg; eluted with CH_2_Cl_2_:MeOH:H_2_O 6:4:0.4) was subjected to CC over Sephadex (MeOH:H_2_O 100:0-90:10) and afforded compounds **9** (3.4 mg), **10** (14.8 mg) and **11** (1.4 mg).

## Results

### Minerals, Nutritional Content, and Extracts Bioactivity

The mineral composition and the nutritional traits of both flowers and leaves of *S. cypria* plants are illustrated on **Table 1**. Flowers appeared to have significantly higher nutritional value than leaves, in terms of protein levels, total fats and carbohydrates, resulting in higher energy content. As for the minerals, flowers are richer in N, P, and Mg, while leaves have higher content of K, Ca, and Na. For the cases of K and Ca, concentration in leaves is almost two times higher than in flowers. Micronutrients as Fe and Cu were found in bigger amounts in leaves when compared to flowers but Zn remained at the same levels in leaves and flowers.

For the evaluation of the extract’s bioactivity (**Table 2**), two different extracts were assayed; the infusion and the decoction of both flowers and leaves. In both cases of extracts, bioactive compounds and the correlated phenolic content appeared significantly higher for the flower extracts. In all cases, the infusion values were higher than the ones obtained from the decoction extracts. Total phenolic content in the infusion of flowers appeared more than double compared to the corresponding leaf extract. The same trend was revealed for all the bioactive compounds (DPPH and FRAP) assays tested, with the most remarkable differences to be evidenced for the total flavonoid content. Here flowers had more than 4 times higher values, in terms of rutin equivalents; 32.004 mg rutin per g of dried tissue for the flowers while the leaf content was found at 7.721 mg rutin per g of dried tissue.

### Essential Oils Yield and Compound Identification

The essential oil yield of the two plant parts tested in this study appeared significantly different. Flowers exhibited higher yield with 0.25% (± SE 0.029) while the essential oils of leaves were in relatively low level and did not exceed 0.03% (± SE 0.003). The GC/MS analysis that followed delivered 34 compounds in the leaves’ oils and 31 compounds in the oils obtained from the flowers; 29 of these compounds appeared in the oils from both plant parts, but with great variability in terms of contribution to the oil profile (**Table 3**). The five and two compounds found only in leaves and flowers respectively, represent components with less than 0.350% (nonanal) each, out of the total chromatogram. In the oils from the leaves, the major terpenes were the monoterpenes hydrocarbons with 51.45%, followed by sesquiterpenes hydrocarbons (23.41%) and oxygenated sesquiterpenes (22.18%), while in flowers the total monoterpenes hydrocarbons reached 88.38%, and oxygenated compounds in total did not exceed 4.35% (1.34% and 2.99%, for mono- and sesquiterpenes, respectively). The major compounds identified in leaves were β-phellandrene (25.11%), β-caryophyllene (22.52%), α-pinene (11.92%), 14 hydroxy-β-caryophyllene (9.88%), and caryophyllene oxide (8.32%). In flowers, the dominant compound is α-pinene (37.97%), followed by β-phellandrene (25.81%), β-pinene (14.67%), β-caryophyllene (6.83%) and sabinene (5.58%). With the exception of β-phellandrene, the other major compounds differ significantly in terms of percentage (%) between the two tested oils. Comparing the total chromatographs of the two tested oils, the majority of the compounds (in terms of percentage participation), are significantly different, except eight compounds (**Table 3**). Considering the essential oil analysis in leaves and flowers separately, this is the first report of the analysis of the essential oils from *S. cypria* flowers.

**Table 3:** Chemical composition (%) of essential oils of *Sideritis cypria* leaves and flowers.

Compound	RI^a^	Leaves	Flowers
α-Thujene	926	0.677 ± 0.016b	0.847 ± 0.002a
α-Pinene	933	11.924 ± 0.29b	37.97 ± 0.294a
Camphene	948	0.018 ± 0.000b	0.084 ± 0.001a
Sabinene	973	5.404 ± 0.090a	5.580 ± 0.017a
β-Pinene	977	4.979 ± 0.091b	14.671 ± 0.05a
β-Myrcene	989	0.773 ± 0.008b	1.016 ± 0.005a
α-Phellandrene	1004	1.891 ± 0.017a	1.577 ± 0.006b
a Terpinene	1017	0.061 ± 0.001a	0.110 ± 0.027a
o Cymene	1024	0.405 ± 0.004a	0.463 ± 0.026a
β-Phellandrene	1029	25.114 ± 0.325a	25.818 ± 0.056a
Eucalyptol	1031	0.017 ± 0.017a	0.042 ± 0.021a
Benzeneacetaldeyhe	1041	0.012 ± 0.006	–
γ-Terpinene	1058	0.153 ± 0.002a	0.205 ± 0.044a
*Cis* Sabinenehydrate	1066	0.095 ± 0.002a	0.083 ± 0.003b
1 octanol	1067	0.049 ± 0.003	–
Terpinolene	1089	0.053 ± 0.001a	0.040 ± 0.003b
*trans* Sabinenehydrate	1100	0.030 ± 0.001a	0.037 ± 0.005a
Nonanal	1104	0.350 ± 0.002	–
α-Campholenal	1127	0.021 ± 0.004b	0.081 ± 0.003a
*trans* Pinocarveol	1139	0.015 ± 0.000b	0.114 ± 0.006a
*trans* Verbenol	1143	0.028 ± 0.002b	0.142 ± 0.007a
Pinocarvone	1163	0.012 ± 0.006b	0.075 ± 0.003a
Terpinen-4-ol	1178	0.130 ± 0.003a	0.140 ± 0.006a
Cryptone	1187	0.160 ± 0.002a	0.069 ± 0.004b
α-Terpineol	1191	0.454 ± 0.008b	0.500 ± 0.008a
Myrtenol	1195	–	0.074 ± 0.005
Cumin aldehyde	1241	0.175 ± 0.002a	0.023 ± 0.001b
Carvone	1244	0.138 ± 0.053	–
Thymol	1299	–	0.034 ± 0.018
β-Caryophyllene	1425	22.525 ± 0.026a	6.836 ± 0.056b
α-Humulene	1462	0.890 ± 0.011a	0.176 ± 0.011b
Caryophyllene oxide	1587	8.325 ± 0.149a	1.498 ± 0.036b
Humulene epoxide	1607	0.177 ± 0.010	–
Camphoric Acid	1636	0.219 ± 0.023a	0.063 ± 0.008b
14 Hydroxy-β- caryophyllene	1666	9.884 ± 0.327a	1.388 ± 0.069b
14 Hydroxy-α-humulene	1707	3.803 ± 0.173a	0.108 ± 0.056b
Total Identified		98.955 ± 0.080	99.864 ± 0.024
Monoterpenes hydrocarbons		51.452 ± 0.836b	88.382 ± 0.240a
Sesquiterpenes hydrocarbons		23.415 ± 0.028a	7.012 ± 0.063b
Oxygenated monoterpenes		1.109 ± 0.048b	1.344 ± 0.043a
Oxygenated sesquiterpenes		22.188 ± 0.658a	2.995 ± 0.158b
Others		0.791 ± 0.027a	0.131 ± 0.010b

a Components listed in order of elution from a Zebron ZB-5 capillary column

^b^values (n=3) in rows followed by the same letter are not significantly different, P ≤ 0.05.

### Identification of Non Volatile Secondary Metabolites

The infusion of the flowers of cultivated *S. cypria* yielded four flavones, isoscutellarein-7-O-[6′″-O-acetyl-β-D-allopyranosyl-(1→2)-β-D-glucopyranoside (**1**) (Lenherr and Mabry, 1987), 4′-O-methyl-isoscutellarein-7-O-[6′″-O-acetyl-β-D-allopyranosyl-(1→2)-β-D-glucopyranoside (**2**) (Venditti et al., 2014), isoscutellarein-7-O-[6′″-O-acetyl-β-D-allopyranosyl-(1→2)]-6″-O-acetyl-β-D-glucopyranoside (**3**) (Halfon et al., 2013), and 4′-O-methyl-hypolaetin-7-O-[6′″-O-acetyl-β-D-allopyranosyl-(1→2)-β-D-glucopyranoside (**4**) (Halfon et al., 2013); four phenylethanoid glucosides, acteoside (**5**) (Kawada et al., 1999), leucosceptoside A (**6**) (Miyase et al., 1982), leonoside A (**8**) (Çaliş et al., 1992), and lamalboside (**9**) (Budzianowski and Skrzypczak, 1995); one iridoid, melittoside (**10**) (Śawia̧tek et al., 1981), and one quinic acid derivative, chlorogenic acid (**11**) (Armata et al., 2008).

**Figure 2:**
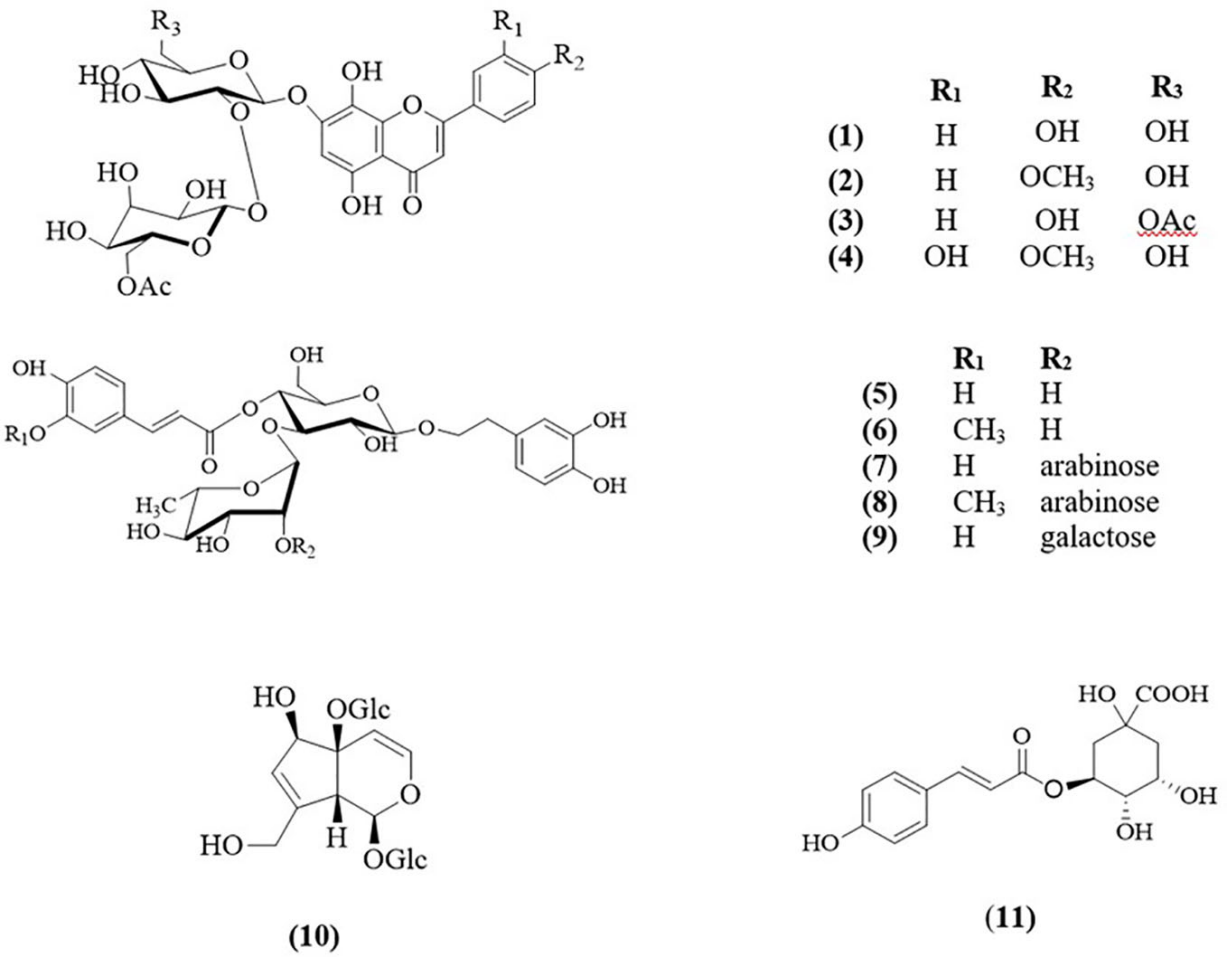
Structures of isolated compounds **1–11**.

Moreover, the infusion of the leaves of the same population afforded two flavones, isoscutellarein-7-O-[6′″-O-acetyl-β-D-allopyranosyl-(1→2)-β-D-glucopyranoside (**1**) and 4′-O-methyl-isoscutellarein-7-O-[6′″-O-acetyl-β-D-allopyranosyl-(1→2)-β-D-glucopyranoside (**2**); five phenylethanoid glucosides, namely the four previously found in the infusion of flowers and lavandulifolioside (**7**) (Başaran et al., 1988), moreover the same iridoid and quinic acid derivative have been isolated (**Figure 2**).

Leonoside A (**8**) was first isolated from *Stachys sieboldii* in 1991 (Nishimura et al., 1991) as stachysoside B. Later, Çaliş et al. (1992) isolated it from *Leonurus glaucescens* and it was given the name “leonoside A”. From the genus *Sideritis* L., it has been previously isolated only from *S. trojana* Bornm. (Kirmizibekmez et al., 2012). Our study confirms the presence of leonoside A in *S. cypria* (Hanoğlu et al., 2020) and we report for the first time the isolation of this compound from the infusion of both, leaves and flowers. It is interesting to point out that compounds **2**- **4**, **9 - 11** had been mentioned previously in the genus *Sideritis* L., but not in *S. cypria*. Thus, the aforementioned compounds are reported as components of *S. cypria* for the first time. The structures of the isolated compounds were elucidated by spectroscopic data. Intriguingly, the NMR data of compounds **8**, **9**, and **10** are not fully recorded in the literature, therefore, herein, are presented (**Tables S1**–**S3**).

It is important to mention that the chemical characterization of the total infusions and the sub fractions were monitored by NMR techniques, which enabled us to identify and isolate all the main and the minor components.

## Discussion

The mineral content of *S. cypria* leaves differs from that of the flowers. As it was mentioned previously, leaves are richer in potassium and calcium while flowers in magnesium and phosphorus, and this is of great importance as reported by Bojovic et al. (2011), as water tea-infusions had large portions of K, P, Na, and Cu, and different parts of the plants can contribute in various ways on human nutrition. Comparing to other species of the genus, *S. cypria* leaves have similar levels of potassium (28.40 g/kg) with the leaves of *S. perfoliata*, ranging from 20.65 to 29.84 g/kg, depending on the cultivation method (Chrysargyris et al., 2019b). *S. cypria* has almost two times higher content of phosphorus and calcium, in both plant parts, than *S. perfoliata* (Chrysargyris et al., 2019b; Lall et al., 2019). Species *S. scardica* and *S. raeseri* appear poorer in terms of mineral content, and demonstrate almost half the quantities of the majority of nutrients as K, P, Ca, Mg (Karapandzova et al., 2013), compared to *S. cypria*. Trikka et al. (2019) evaluated different species of *Sideritis* genus from different geographical regions of Greece and plants exhibited high variability, even within species (*S. perfoliata, S. scarica, S. raeseri*), and had lower contents in minerals than *S. cypria*. These variations may be the effect of the cultivation practices applied or/and the harvesting periods (Chrysargyris et al., 2019b).

Regarding the nutritional value, the flowers of *S. cypria* are richer in protein, fats, and carbohydrates than leaves. *S. perfoliata* aerial parts (leaves) demonstrate protein content up to 14.64%, while in *S. cypria* the corresponding content was 8.06% and 9.02% for leaves and flowers. Total fats and carbohydrates demonstrated almost similar values for *S. cypria* (1.78% and 72.90%) and *S. perfoliata* (1.76% and 73.53%). A research conducted to investigate the chemical profile and metabolite content of *S. italica*, revealed that the protein content of the plant parts were 6% and 10% for the leaves and flowers, respectively (Menghini et al., 2014), values similar to *S. cypria* protein content. Generally, in the Lamiaceae family, protein content may vary among different species of the same genus. As it was assayed by Tunçtürk et al. (2017), *Thymus* species had protein content varying from 6.85-14.34%, while *Mentha* species as *M. longifolia* and *M. spicata* demonstrated protein content of 13.86% and 15.13%, respectively.

The bioactive substances of the two types of aqueous extracts for both plant parts demonstrated the same trend; flowers are richer in compounds as phenolics and flavonoids, and thus, exhibiting higher bioactivity (Phatak and Hendre, 2014). Additionally, the infusions demonstrated higher bioactivity and appear to be richer than decoctions, due to the higher polarity of the solvent used (Martins et al., 2015). In general, plant’s extracts appear to be less strong compared to other species of genus. *S. syriaca*’s decoction demonstrated total phenolic compounds almost 18 mg GAE/g Dw (Goulas et al., 2014), while *S. cypria* decoction ranged between 1.39 and 3.92 mg GAE/g Dw (for leaves and flowers, respectively). Nevertheless, plant species, cultivation practices, environmental conditions, and extraction methodology are parameters, to name a few, that affect the bioactive status of plants (González-Burgos et al., 2011; Chrysargyris et al., 2019b).

According to literature, *Sideritis* genus species yield low essential oil quantities, compared to other Lamiaceae species (González-Burgos et al., 2011). There are reports that indicate this yield variation among species, that range from traces (< 0.01%) of essential oil in many *Sideritis* species (Kirimer et al., 2004; Bojovic et al., 2011) to 0.94% (*S. lanata)* (Koutsaviti et al., 2013). *S. cypria* follows the same trend, with the flower’s essential oil yield to be significantly higher (0.25%) than leaves’ yield, which was measured at considerably low numbers of 0.03%. Hanoğlu et al. (2016; 2020) recorded a yield of 0.49%, when hydrodistilled *S. cypria* plants, during or post-flowering that were collected from mountains in north Cyprus (500–750 m altitude). This variation with the present results might be attributed that wild harvested plant tissue in mountain areas (Hanoğlu et al., 2016; Hanoğlu et al., 2020) can differ on the essential oil yield and aromatic profile, compared with cultivated crop in lowland.

As for the compounds, of the two oils tested (leaves and flowers), analysis showed remarkable differences in composition. The major compounds of leaves are β-phellandrene (25.11%) and β-caryophyllene (22.52%), followed by α-pinene (11.92%), 14 hydroxy-β-caryophyllene (9.88%), and caryophyllene oxide (8.32%). All these components were identified as well by previous studies of the plant during flowering phase, but with different percentages, while β-phellanderene was identified again as the major compound (17.83%) (Hanoğlu et al., 2020). The same authors referenced their previous work (Hanoğlu et al., 2016) mentioning that when oils from post flowering stage were analyzed, the compounds and their amounts varied, compared to those during flowering. Plants evaluated in this study were collected during flowering stage, and leaves’ oil profile is similar to the report of Hanoğlu et al. (2020), but with differences mostly in secondary compounds, in terms of their participation in the oil profile. These differences can be ascribed to the separate analysis of leaves and flowers, and to the differences that occur due to plant’s origin and cultivation practices. Hanoğlu et al. (2020) collected plants from the mountains of north Cyprus, while plants tested in this study are plants from the first official cultivation reported in Cyprus for *S. cypria*. That means that plants are subjected to specific irrigation regimes and cultivation practices, while plants collected from wild exhibit great variability. Todorova and Trendafilova (2014) reviewed this variation in other *Sideritis* species oils as well, mentioning region as a factor of this variation. Chrysargyris et al. (2019b) also reported that cultivation practices and harvesting periods affect components of *Sideritis* species oils.

To our knowledge, this is the first report of *S. cypria* oils analysis that separates oils from different plant parts, and is the first analysis of the flower oil. The major compounds found in flower oil are α-pinene (37.97%), β-phellandrene (25.81%), β-pinene (14.67%), and β-caryophyllene (8.83%). These differences between flowers and leaves, concern not only single components, but can be examined as differences in the grouped compounds, as leaves appear richer in sesquiterpenes hydrocarbons (23.41%) and oxygenated sesquiterpenes (22.18%), while in flower the corresponding percentages are together almost 10% (7.02% and 2.99%, respectively). This is of great importance, as consumer can use either leaves or flowers or mixture of them during their usage of *S. cypria*, reflecting different aromatic profile and properties. Bojovic et al. (2011) reviewed the phytochemical profile of *Sideritis* species, indicating that essential oils are characterised by high contents of monoterpene hydrocarbons with α-pinene, β-pinene, sabinene, myrcene or limonene and of sesquiterpene hydrocarbons, particularly δ-cadinene and β-caryophyllene, as the main compounds. However, *S. cypria* is not on that extensive review list of species, highlighting the importance of the present study.

Monoterpenes such as pinenes and phellandrenes, among others are the main components of the odour of medicinal plants with biocidal activities. For example, β-phellandrene has been tested as a strong antimicrobial terpene while it exhibits insect repellent activities (Harrewijn et al., 2001). It has shown *in vitro* activity against *Bacillus* sp.*, Candida albicans, Escherichia coli, Pseudomonas aeruginosa, and S. aureus* (Zhang et al., 2017). In plants, pinenes show fungicidal activity and have been used for centuries to produce flavors and fragrances, while the antimicrobial activity of some essential oils is attributed to the presence of α- and/or β-pinene (Rivas da Silva et al., 2012). α-Pinene is a monoterpene and member of hydrocarbon group of bicyclic terpenes that has not only a wide range of uses as a food additive (Yang et al., 2016), but also exhibits great biological activities as insecticidal, spasmolytic, anti-listerial, and anticholinesterase (Orhan et al., 2006). Additionally, Akutsu et al. (2002), found that α-pinene has a potential anti-stress activity. Moreover, β-pinene has also exhibited antidepressant activity, and furthermore it’s mode of action could be connected to the mechanism of the action of the most frequently used antidepressant drugs (Guzman-Guttierez et al., 2015). Orhan et al. (2006) also reported the anti-inflammatory activity of the compound, while reviewing the synergistic effect of α-pinene with β-caryophyllene, as anti- inflammatory compounds. Interestingly, β-caryophyllene alone is a compound which has been demonstrated to possess a great potential application for various pathological conditions, as central nervous system diseases (Parkinson’s disease, Alzheimer’s disease), osteoporosis, cancer, and antibacterial (Francomano et al., 2019). Importantly, it is also approved as food additive, taste enhancer, and flavoring agent by U.S. Food and Drug Administration (FDA) and European Food Safety Authority (EFSA) (Fidyt et al., 2016; Machado et al., 2018).

Continuing our study on *Sideritis* genus, this work reports for the first time the composition of the traditional infusions of *S. cypria* Post. The genus *Sideritis* L. is characterized by the presence of several different secondary metabolites, mainly terpenes and various phenolic derivatives. In the present study, overall, eleven compounds were isolated from both infusions of the flowers and the leaves of cultivated *S. cypria*. It is interesting to note that the two plant parts showed similar chemical composition. Precisely, from the sample of the flowers were isolated more flavones, compared to the sample of the leaves in which were identified more phenylethanoid glucosides.

Numerous studies over the last years have revealed the presence of a great number of glycosides of 8-hydroxy flavones in *Sideritis* species, such as acetylated or non-acetylated 7-O-allosyl (1→2)glucosides of isoscutellarein (8-hydroxy apigenin) and hypolaetin (8-hydroxy luteolin), as well as their 4′-methoxy derivatives (EMA, 2015; Stanoeva et al., 2015). In the current study, three isoscutellarein and one hypolaetin derivatives were found. The results are in agreement with previously well studied *Sideritis* species belonging to the section *Empedoclia*, i.e., *S. scardica* (Todorova and Trendafilova, 2014; EMA, 2015), *S. perfoliata* L. subsp. *perfoliata* (Charami et al., 2008; Chrysargyris et al., 2019b), *S. euboea* (Tomou et al., 2019), *S. raeseri* (Gabrieli et al., 2005; Romanucci et al., 2017), and *S. syriaca* (Plioukas et al., 2010; Goulas et al., 2014).

Hanoğlu et al. (2020) studied the chemical composition of non polar and polar extracts of *S. cypria* wild populations and reported the presence of four flavones, four phenylethanoid glucosides and one iridoid glucoside in the polar extracts. Comparing our results to the literature data, we confirm the presence of the same phenylethanoid glucosides, with the only difference the isolation of lamalboside in both infusions. It is noteworthy that lamalboside has been isolated previously only from two *Sideritis* species belonging to the *Empedoclia* section; *S. germanicopolitana* Bornm. and *S. trojana* Bornm. (Kirmizibekmez et al., 2012; Kirmizibekmez et al., 2019). Lamalboside, also known as lamiuside A, was first isolated and described from the genus *Lamium* L. (Budzianowski and Skrzypczak, 1995; Ito et al., 2006; Yalçın et al., 2007). Furthermore, considering the identification of the flavonoid derivatives, the two infusions were characterized by isoscutellarein derivatives. However, Hanoğlu et al. (2020) reported the isolation of four flavones; one isoscutellarein derivative and three apigenin derivatives. This distinguishment could be attributed not only in the fact that our plant material is originated from cultivation, but also to the different polarity of the investigated samples between the two studies. Importantly, our research also revealed the presence of chlorogenic acid, which has been previously found in many *Sideritis* spp. (Armata et al., 2008; Samanidou et al., 2012; Goulas et al., 2014; Irakli et al., 2018; Chrysargyris et al., 2019b; Tomou et al., 2019). It is interesting to note that in both infusions the main iridoid derivative was melittoside. In the specific section (*Empedoclia*), melittoside have been reported in the species *S. euboea* Heldr. (Tomou et al., 2019), *S. montana* L. (Koleva et al., 2003; Fraga, 2012), *S. montana* L. subsp. *montana* (Venditti et al., 2016), *S. germanicopolitana* Bornm (Kirmizibekmez et al., 2019), *S. perfoliata* L. subsp. *perfoliata* (Chrysargyris et al., 2019b), and *S. syriaca* L. (Koleva et al., 2003; Fraga, 2012). Acteoside and leucosceptoside A, found in our study, have been also isolated from *S. euboea* Heldr. (Tomou et al., 2019), *S. perfoliata* L. subsp. *perfoliata* (Charami et al., 2008; Chrysargyris et al., 2019b), *S. scardica* Gris. (Fraga, 2012; Todorova and Trendafilova, 2014), *S. lysia* Boiss et. Heldr. (Fraga, 2012), and *S. raeseri* Boiss et. Heldr. (Petreska et al., 2011), while lavandulifolioside has been isolated from *S. euboea* Heldr. (Tomou et al., 2019), *S. perfoliata* L. subsp. *perfoliata* (Charami et al., 2008; Chrysargyris et al., 2019b), and *S. lysia* Boiss et. Heldr (Fraga, 2012). Moreover, a survey of four infusions of cultivated *S. raeseri* subsp. *raeseri* was carried out and underlay the great antioxidant activity due to the high phenolic content, including chlorogenic acid, phenylethanoid glucosides and flavonoid derivatives (Pljevljakušić et al., 2011).

Chrysargyris and co-workers (2019b) have revealed the presence of three isoscutellarein derivatives, two phenylethanoids, one quinic acid derivative and six iridoids from the infusion of the cultivated *S. perfoliata* L. subsp. *perfoliata* from Cyprus. Of note, our results are in accordance with this study. It is interesting to point out that cultivated *S. cypria* is very poor in iridoids compared to cultivated *S. perfoliata* L. subsp. *perfoliata*, while on the other hand *S. cypria* consists more phenylethanoid glucosides. In the context of the presence of phenylethanoid glucosides, bearing three sugar moieties, are common group of secondary metabolites of this genus, but the trisaccharide derivatives with galactose as one of the sugars are rare.

Several studies have mentioned the antioxidant activity of isoscutellarein and hypolaetin derivatives (Gabrieli et al., 2005; Armata et al., 2008; Charami et al., 2008; Kirmizibekmez et al., 2012). It is well established that flavonoids with at least one hydroxyl-group in B ring exhibit high antioxidant activity (Charami et al., 2008; Sarian et al., 2017). However, the existence of a hydroxyl group in ring A and catechol structure or 4′-hydroxyl group in ring B improve more the antioxidant activity (Charami et al., 2008; Sarian et al., 2017). Furthermore, phenylethanoid glucosides are known for various pharmacological activities (Fu et al., 2008). Precisely, Charami et al. (2008) reported the antioxidant and anti-inflammatory activity of some isolated phenylethanoid glucosides and flavones derivatives from the species *S. perfoliata* subsp. *perfoliata*. Phenylethanoid glucosides exhibited better anti-inflammatory activity, compared to flavonoids due to their structures (caffeoyl group and to their *o*-dihydroxyphenyl group) (Charami et al., 2008). Moreover, a survey of four infusions of cultivated *S. raeseri* subsp. *raeseri* was carried out and underlay the great antioxidant activity due to the high phenolic content, including chlorogenic acid, phenylethanoid glucosides, and flavonoid derivatives (Pljevljakušić et al., 2011). Therefore, we could assume that the presence of flavones derivatives in combination with the abundance of phenylethanoid glucosides and chlorogenic acid in our samples could enhance the pharmacological activity, as well as it could justify the ethnopharmacological uses of *S. cypria*.

The present study revealed the chemical variation of the flowers and leaves essential oils (EOs) in comparison to the wild population of *S. cypria* previously investigated (Hanoğlu et al., 2016). The main constituents of the aerial parts of wild *S. cypria* EO were epi-cubebol (11.9%), *trans*-piperitol (8.9%), α-pinene (4.3%), and β-pinene (3.6%), while the main constituents of the flowers and leaves EOs were α-/β-pinenes in significant higher concentration, sabinene, β-phellandrene (ca. 25% in both EOs), β-caryophyllene, caryophyllene oxide, and 14-hydroxy-β-caryophyllene. In accordance with the EOs of Greek *Sideritis* species, where monoterpenes are the dominant components (Aligiannis et al., 2001; González-Burgos et al., 2011; Kloukina et al., 2019), the present samples are also rich in monoterpene hydrocarbons followed by sesquiterpenes. We assume that different factors (altitude, cultivation methods, and distillation methods) play an important role on the observed chemical variations.

## Conclusion

The present study illustrates the phytochemical investigation of leaves and flowers of cultivated *S. cypria*, is a very rare and vulnerable species endemic to Cyprus with a small distribution area restricted to the Pentadaktylos Range (Tsintides et al., 2007; Bilz, 2011). The present study reveals that the cultivation of *S. cypria* is feasible without affecting its chemical profile. Cyprus ironwort has a chemical profile rich in bioactive secondary metabolites and can be used as culinary herb, but the plant material sold in local markets should not be collected from the field and should come from crops. *S. cypria* has a high phosphorus and calcium levels in both leaves and flowers. The nutritional value is mainly oriented on flowers which are richer in protein, fats, and carbohydrates than leaves. Flowers exhibited higher essential oil yield comparing to the leaves. The five major constituents identified in leaves were β-phellandrene, β-caryophyllene, α-pinene, 14 hydroxy-β-caryophyllene, and caryophyllene oxide, while in flowers the dominant compound was α-pinene, β-phellandrene, β-pinene, β-caryophyllene, and sabinene. Moreover, the infusion of the flowers was more abundant in flavones, though the infusion of the leaves was mainly consisted by phenylethanoid glucosides, based on the isolated compounds and NMR data.

## Data Availability Statement

All datasets generated for this study are included in the article/**Supplementary Material**.

## Author Contributions

KL carried out the chemical analyses of the infusions. E-MT contributed to the chemical analyses of the infusions and to the writing of the article. HS supervised the chemical analyses of the infusions and contributed to the writing of the article. AC and CD carried out the analyses of EOs, of mineral and nutritional contents of the leaves and flowers infusions/decoctions. NT supervised the latter analyses and contributed to the writing of the article.

## Funding

The research performed at the Department of Pharmacognosy & Chemistry of Natural Products and at the Department of Agricultural Sciences, Biotechnology and Food Science, were funded by the National & Kapodistrian University of Athens and by the Cyprus University of Technology, respectively. Funding for publication was granted by the Cyprus University of Technology Open Access Author Fund.

## Conflict of Interest

The authors declare that the research was conducted in the absence of any commercial or financial relationships that could be construed as a potential conflict of interest.
