# Supplementary material for: Traditionally Used Sideritis cypria Post.: Phytochemistry, Nutritional Content, Bioactive Compounds of Cultivated Populations
Source: Front Pharmacol. 2020 May 12;11:650. doi: 10.3389/fphar.2020.00650 (PMC7235332; doi:10.3389/fphar.2020.00650)
Supplement: Supplementary file 1 [file DataSheet_1.docx]

**Supplementary material**

**Traditionally used *Sideritis cypria* Post.: Phytochemistry, nutritional content, bioactive compounds of cultivated populations**

Krystalia Lytra^1^, Ekaterina-Michaela Tomou^1^, Antonios Chrysargyris^2^, Chryssoula Drouza^2^, Helen Skaltsa^1^*, Nikolaos Tzortzakis^2^*

**Table S1:** ^1^H-NMR data of compound **8** (CD_3_OD)

**Table S2:** 1D- and 2D- NMR data of compound **9** (CD_3_OD)

**Table S3:** 1D- and 2D- NMR data of compound **10** (CD_3_OD).

Compound **8**


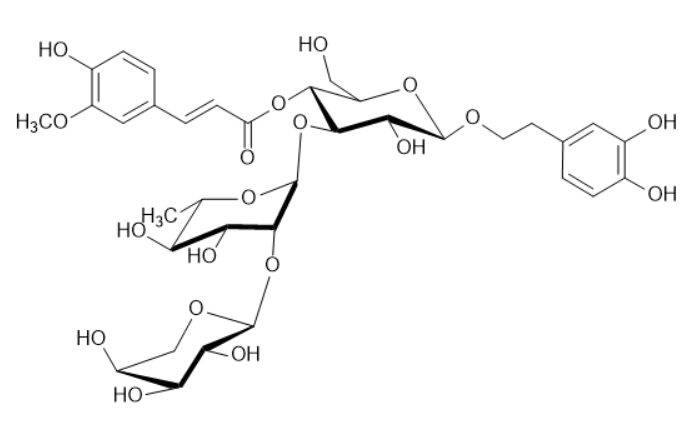


**Table S1:** ^1^H-NMR data of compound **8** (CD_3_OD)

| **Position** | **δ_H_ (ppm, *J* in Hz)** |
| --- | --- |
| **aglycone** | |
| 1 | - |
| 2 | 6.71 (d, *J*=2.0) |
| 3 | - |
| 4 | - |
| 5 | 6.68 (d, *J*=8.0) |
| 6 | 6.57 (dd, *J*=8.0/ 2.0) |
| 7 | 2.80 (t, *J*=7.2) |
| 8a | 4.05 (m) |
| 8b | 3.72 (m) |
| **glucose** | |
| 1' | 4.38 (d, *J*=7.9) |
| 2' | 3.39 (t, *J*=7.9) |
| 3' | * |
| 4' | 4.92^a^ |
| 5' | 3.49 (m) |
| 6a' | 3.65(*) |
| 6b' | 3.53(*) |
| **rhamnose** | |
| 1'' | 5.50 (d, *J*=1.7) |
| 2'' | 3.95 (dd, *J*=3.4/1.7) |
| 3'' | 3.64 (m) |
| 4'' | 3.29 (*) |
| 5'' | 3.58 (m) |
| 6'' | 1.07 (d, *J*=6.3) |
| **arabinose** | |
| 1''' | 4.31 (d, *J*=7.4) |
| 2''' | 3.60 (m) |
| 3''' | 3.51(*) |
| 4''' | 3.77 (brs) |
| 5a''' | 3.86(*) |
| 5b''' | 3.54(*) |
| **feruloyl group** | |
| 1'''' | - |
| 2'''' | 7.21 (d, *J*=1.8) |
| 3'''' | - |
| 4'''' | - |
| 5'''' | 6.82 (d, *J*=8.1) |
| 6'''' | 7.10 (dd, *J*=8.1,1.8) |
| 7'''' | 7.67 (d, *J*=15.9) |
| 8'''' | 6.38 (d, *J*=15.9) |
| OMe | 3.90 (s) |

^a^ overlapped by the signal of the solvent; *assignments overlapped

Compound **9**


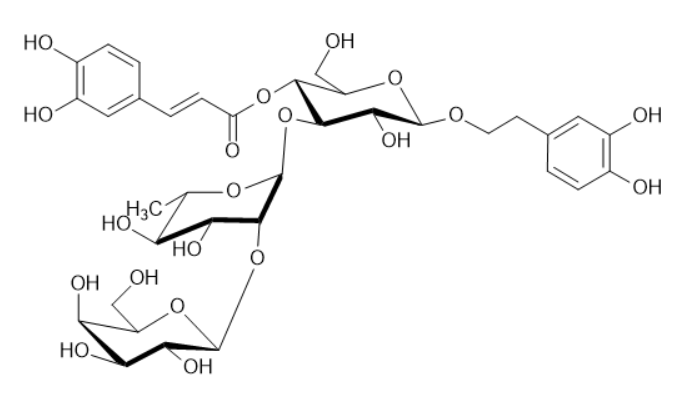


**Table S2:** 1D- and 2D- NMR data of compound **9** (CD_3_OD)

*assignments overlapped

| **Position** | **δ_c_ (ppm)** | **δ_H_ (ppm, *J* in Hz)** | **HMBC** |
| --- | --- | --- | --- |
| **aglycone** | | | |
| 1 | 131.3 | - | - |
| 2 | 117.0 | 6.71 (d, *J*=1.9) | C-1, C-3, C-4, C-6, C-7 |
| 3 | 144.2 | - | - |
| 4 | 146.3 | - | - |
| 5 | 116.9 | 6.69 (d, *J*=8.0) | C-1, C-3, C-4 |
| 6 | 121.1 | 6.58 (dd, *J*=8.0/1.9) | C-3, C-5 |
| 7 | 36.5 | 2.80 (t, *J*=7.4) | C-1, C-2, C-6, C-8 |
| 8a | 72.0 | 4.07 (m) | C-1 |
| 8b |  | 3.74 (m) | C-1 |
| **glucose** | | | |
| 1' | 104.1 | 4.38 (d, *J*=7.9) | C-3', C-8 |
| 2' | 75.5 | 3.39 (t, *J­*=7.9) |  |
| 3' | 82.3 | 3.77 (*) | C-1'' |
| 4' | 69.9 | 4.93 (t, *J*=8.9) | C-5', C-6', C-9'''' |
| 5' | 75.9 | 3.53 (*) |  |
| 6a' | 62.0 | 3.61 (dd, *J*=11.0/*) |  |
| 6b' |  | 3.54 (*) |  |
| **rhamnose** | | | |
| 1'' | 102.0 | 5.57 (brs) | C-3', C-3'' |
| 2'' | 82.0 | 3.99 (dd, *J*=3.3/1.2) |  |
| 3'' | 71.8 | 3.65 (dd, *J*=9.5/3.3) |  |
| 4'' | 74.0 | 3.28 (t, *J*=9.5) | C-6'' |
| 5'' | 69.6 | 3.54 (*) |  |
| 6'' | 18.6 | 1.04 (d, *J*=6.0) | C-4'', C-5'' |
| **galactose** | | | |
| 1''' | 107.2 | 4.35 (d, *J_=_*7.4) | C-2'' |
| 2''' | 72.5 | 3.57 (*) |  |
| 3''' | 74.4 | 3.47 (dd, *J*=9.9/3.3) |  |
| 4''' | 69.9 | 3.80 (t, *J*=3.3) |  |
| 5''' | 76.1 | 3.52 (*) |  |
| 6a''' | 62.3 | 3.81 (dd, *J*=11.1/*) |  |
| 6b''' |  | 3.72 (dd, *J*=11.1/*) |  |
| **caffeoyl group** | | | |
| 1'''' | 127.7 | - | - |
| 2'''' | 115.0 | 7.07 (d, *J*=1.9) | C-6'''', C-7'''', C-3'''' |
| 3'''' | 149.9 | - | - |
| 4'''' | 146.5 | - | - |
| 5'''' | 116.2 | 6.79 (d, *J*=8.2) | C-2'''', C-6'''', C-1'''', C-4'''', C-3'''' |
| 6'''' | 122.9 | 6.97 (dd, *J*=8.2/1.9) | C-2'''', C-5'''', C-7'''', C-3'''' |
| 7'''' | 147.8 | 7.61 (d, *J*=15.9) | C-2'''', C-6'''', C-1'''', C-9'''' |
| 8'''' | 114.5 | 6.28 (d, *J*=15.9) | C-1'''', C-9'''' |
| 9'''' | 168.6 | - | - |

Compound **10**


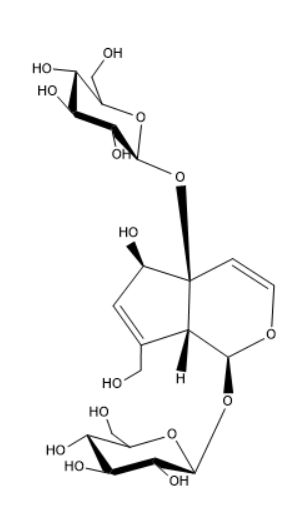


**Table S3:** 1D- and 2D- NMR data of compound **10** (CD_3_OD).

| **Position** | **δ_c_ (ppm)** | | **δ_H_ (ppm, *J* in Hz)** | **HMBC** |
| --- | --- | --- | --- | --- |
| **aglycone** | | | | |
| 1 | 93.6 | | 5.59 (d, *J*=3.9) | C-1', C-3, C-5, C-8 |
| 2 | - | | - | - |
| 3 | 143.1 | | 6.37 (d, *J=*6.5) | C-1, C-4, C-5 |
| 4 | 105.0 | | 5.12 (d, *J*=6.5) | C-3, C-5, C-9 |
| 5 | 79.7 | | - | - |
| 6 | 79.4 | | 4.37 (brs) | C-4, C-7, C-8, C-9 |
| 7 | 128.0 | | 5.79 (brs) | C-5, C-8, C-9 |
| 8 | 146.9 | | - | - |
| 9 | 51.0 | | 3.33 (*) |  |
| 10 60.4 | | 4.20 (d, *J*=7.6) | | C-7, C-8 |
| **glucose** | | | | |
| 1' | 97.9 | | 4.61 (d, *J*=7.9) | C-1 |
| 2' | 74.4 | | 3.26 (t, *J*=7.9) |  |
| 3', 4', 5' | * | | 3.60-3.33 (*) |  |
| 6a', 6b' | 61.5/62.8 | | 3.79-3.63 (*) |  |
| **glucose** | | | | |
| 1'' | 99.5 | | 4.67 (d, *J*=7.9) | C-5 |
| 2'' | 71.1 | | 3.29^a^ |  |
| 3'', 4'', 5'' | * | | 3.60-3.33 (*) |  |
| 6a'', 6b'' | 61.5/62.8 | | 3.79-3.63 (*) |  |

^a^ overlapped by the signal of the solvent;*assignments overlapped
